# Networked computing systems for bio-diversity and environmental preservation

**DOI:** 10.1038/s41598-022-07226-z

**Published:** 2022-02-28

**Authors:** A. A. Periola, A. A. Alonge, K. A. Ogudo

**Affiliations:** Electrical and Electronic Engineering Technology, University of Johannesburg, Johannesburg, South Africa

**Keywords:** Engineering, Electrical and electronic engineering

## Abstract

Computing platforms have a high water footprint that poses threat to biodiversity preservation. The high water footprint reduces water availability for habitat preservation. Hence, approaches that reduce the water footprint are needful. The presented research proposes an approach that reduces the need for water in future computing platforms. It proposes a hybrid computing platform that comprises terrestrial and non-terrestrial computing platforms. The performance benefit of using hybrid computing platforms is evaluated using the novel water potential metric. The water potential (WP) quantifies the need for water (for cooling) by computing platforms. A low WP shows that computing platforms have reduced the need for water and indicates better performance than a high WP from the perspective of reducing water footprint. Evaluation is done via performance formulation and stochastic simulation of the WP metric. Analysis shows that using the hybrid computing platform instead of the existing approach that utilizes only water-cooled terrestrial data centres reduces the WP by (4.9–93) % on average.

## Introduction

Data centres have a high water footprint^1,2^ and play an important role on the internet. They can also be sited in the ocean^3,4^ and space^5,6^. Reducing data centre water footprint is crucial to improve water security and enable environment-friendly operation. In addition, cloud operators have developed different approaches to reduce data centre water footprint. This has led to emphasizing metrics and events like water footprint^7^, stratosphere cooling^8^ and water usage effectiveness^9,10^.

The need to realize environment-friendly data centre operation should consider the use of terrestrial and non-terrestrial computing platforms. This should be done while complying with data retention policies that influence data access in cloud computing^11,12^. This can be realized via a network architecture that considers terrestrial, stratosphere based, outer space-based and underwater data centres.

The motivation for the presented research is designing solutions and network architecture enabling environment-friendly operation of computing platforms in future context and considering hybrid computing platforms. Hybrid computing platforms comprise terrestrial and non-terrestrial computing platforms. The non-terrestrial computing platforms do not require terrestrial water resources for cooling. Hence, their inclusion in hybrid computing platforms reduces water footprint and makes more water available for sustaining different habitats. The challenge being addressed is that of ensuring an environment-friendly operation (habitat preservation) of hybrid computing platforms while complying with data sovereignty policies. This is done by reducing the cloud computing water footprint. The consideration focuses on hybrid computing platforms and advances existing work that focuses on terrestrial computing platforms. In addition, existing work in^1^ shows that the evaluation of water use by data centres requires having access to data on the water footprint. However, it is important to devise an approach that can enable the determination of the amount of water to be used by a data centre (especially in cooling). The discussion here addresses this challenge. The contributions of the paper are:

1. First, the paper presents a sustainable model for a hybrid cloud computing platform. The hybrid cloud computing platform comprises water-cooled terrestrial, stratosphere-based, space-based and underwater data centres. The model considers the realization of sustainable operation and complying with data retention policies.
2. Second, the paper proposes a network architecture that meets the goals of environment-friendly operation and complying with data retention policies. It identifies the water potential (WP) as a metric that describes the need to use water aboard a data centre for cooling. A low WP is more beneficial than a high WP. This is because a low WP implies that the data centres require less water for cooling while a high WP describes data centres requiring more water for cooling. The WP is formulated for the existing case (with only terrestrial data centres) and the proposed case (hybrid computing platform).
3. Third, the paper develops a mathematical model that formulates the water potential (WP) as the main performance metric. The WP is formulated for the existing case (with only terrestrial data centre) and the proposed case (hybrid computing platform). In the existing case, the WP is formulated considering terrestrial data centres that are utilizing water-cooling or air-cooling methods. The formulation of the WP also incorporates the number of cooling stages in a given data centre as an important parameter. In this case, data centres being considered can either be in terrestrial or non-terrestrial locations. This considers the scenario in the existing case (comprising only terrestrial data centres) and the proposed case (hybrid computing platform). In addition, the formulation of the WP in the proposed case (hybrid computing platform) considers scenarios where the hybrid computing platform comprises different combinations of computing platforms in varying locations.
4. Fourth, the paper conducts stochastic simulations to investigate how the use of the proposed mechanism reduces the WP. The analysis is done considering different cases where terrestrial computing platforms are used alongside space, stratosphere or underwater data centres.

The path for the presented research starts from the design of a network architecture that describes relations between terrestrial computing platforms and non-terrestrial computing platforms. The non-terrestrial computing platforms do not need terrestrial water resources for cooling. The need for water is also recognized by the computing platforms and is formulated using the WP. The WP is formulated considering different cases where terrestrial computing platforms are combined with non-terrestrial computing platforms (in different locations). Evaluation of the WP in the proposed case and the existing case is done via stochastic simulation to prevent a greedy evaluation approach.

The research proceeds in the following manner. There is an introduction i.e. “Introduction” section that is followed by a discussion on existing work i.e. “Background work” section. There are three aspects related to the methods. These are the description of solutions (“Solution—realizing sustainability” section) and performance results (“Performance formulation” section). In addition, the discussion in “Performance simulation and evaluation” section discusses the results of performance evaluation. “Conclusion” section is the conclusion.

## Background work

Service providers such as Google and Microsoft utilize water for cooling data centres as seen in^1^. The important role of metrics such as the WUE is recognized. It is also observed that only a handful of organizations such as Facebook make the information on their WUE publicly available.

However, Google and Microsoft provide data on the total water footprint (WF). It is also observed that Google handles the WF confidentially. The realization of sustainability requires having access to information on the WF, water source and the WUE. However, water source and WF information have been observed to be confidentially held by service providers operating large scale cloud computing platforms.

Kass et al*.*^2^ note the need for achieving sustainable computing in the evolving global connected era. It is recognized that cloud service providers need to use new metrics for evaluating environmental sustainability. The focus has been on the use of metrics such as power usage effectiveness (PUE), WUE, Network usage effectiveness, and land usage effectiveness (LUE). Current data centres have a high level of resource usage effectiveness (RUE). The RUE comprises energy usage, water usage and land usage. The metrics of PUE, and {WUE, WF} incorporate energy usage and water usage, respectively. The sustainable data centre should have a PUE and WUE that is close to 1.00 and 0.00, respectively. However, additional consideration on the LUE is required.

The use of underwater data centres has received research attention^3,4^. Hume in^4^ notes that using underwater data centres has the benefits of low cooling and access costs for ocean utilization. From the perspective of quality of service (QoS), the use of underwater data centres reduces content access latency for coastal subscribers. The discussion in^4^ recognises that bio-growth poses a challenge to underwater data centre operators. This observation is made from the perspective of using a small number of underwater data centres. Such a consideration is useful if the underwater data centre is used for special computing cases. However, the case where a significant number of underwater data centres are deployed should be considered. In such a case, the effect of temperature increase on aquatic biodiversity becomes important.

Brown in^5^ discusses the benefits of using underwater data centres. These are low estate acquisition costs, reduced bureaucracy and use of low-cost renewable energy. Like^4^, the discussion in^5^ recognizes the ocean’s suitability as a data centre heat sink. However, the effect of a significant rise in underwater temperature due to the use of underwater data centres has not been considered.

The consideration of sustainable data centre operation is necessary to preserve the surrounding terrestrial habitat. This concept applies to the avian, terrestrial and underwater habitats that provide living space for different life forms. The release of heat from data centres in these locations into the corresponding habitats influences the existence of these life forms. Hence, the realization of sustainable data centre operation is important. Non-terrestrial data centres should be considered since data centres can be deployed in the stratosphere. In this location, they can benefit from free stratospheric cooling^8^.

Jones in^9^ recognises the need to reduce data centre high water and energy consumption. Different approaches that can be used to reduce data centre resource consumption are identified. Examples of such techniques are removing the compression chillers and cooling towers, leveraging on free air-cooling, warm water cooling, and the use of artificial intelligence (e.g. Google DeepMind). In addition, resources such as energy and water resources are recognized to be used during server idle epochs. From the perspective of^9^, resource consumption can be reduced by identifying servers that are idle to reduce their resource usage and also use warm water cooling.

Facebook discusses the technologies such as renewable energy, machine learning and minimizing water use being deployed to realize sustainable data centre operations^10^. The discussion focuses on minimizing the natural resources being used by data centres. Resource usage reduction in this manner lowers data centre operational costs. The discussion in^10,11^ links increased energy usage with greenhouse gas emissions, climate change and biosphere variability in data centre locations. A change in the biosphere affects the living habitat. In this context, the biosphere comprises terrestrial, underwater and avian habitats. However, the anthropogenic effect arising from pollution due to data centre cooling causes changes in aquatic and avian biodiversity^12,13^ requires additional consideration.

It is recognized that there is an increasing awareness of the need to reduce data centre resource consumption especially with regards to energy and water. An important resource that is also recognized is land whose use is determined by the LUE. It is also recognized that big cloud computing service providers such as Amazon and Microsoft may be unwilling to share detailed information on water use. It is also recognized that greenhouse gas emissions from data centre operations induce climate change thereby causing changes in living habitats. However, a mechanism to ensure that anthropogenic effects resulting from data centre operation do not cause habitat extinction and biodiversity degradation is yet to receive sufficient research consideration.

Futhermore, it is observed that data centre sustainability is separately sought by different cloud providers. Hence, there is no mechanism enabling users (or organizations) to make the best decision as regards realizing sustainable computing across different vendors with data centres in different locations. In this case, subscribers consider the choice of computing platform during the process of algorithm execution and data processing. Currently, this is not achievable as cloud vendors don’t inter-operate their server farms in the computing platform facilities.

Nevertheless, cloud computing platforms provide an invaluable resource for acquiring and processing data related to environmental biodiversity. In this regard, cloud computing platforms play an important role in techno-environment analysis related to bio-diversity as seen in^14,15^.

Ganchev^14^ describe the use of underwater and terrestrial networks for biodiversity monitoring in the marine and underwater environment, respectively. In the monitoring process, acoustic signals are used to evaluate biodiversity assessment. The acoustic signals are transmitted from the underwater and terrestrial environment to a data centre via a 3G/4G network. Users can also access the acoustic signal being transmitted via the internet.

In monitoring the terrestrial environment, acoustic signals are acquired from a remote station comprising a radio transceiver at a local remote switch that constitutes a base station. The base station transmits data to the storage and processing platform. The ARBIMON II project is recognized to benefit from advances in cloud computing applications. The focus of^14^ is describing a network system that utilizes acoustic signals for monitoring biodiversity in a terrestrial and underwater habitat. The proposed network utilizes terrestrial networks (3G/4G networks) for acoustic signal aggregation and acquisition. In the context of the proposed research, cloud computing platforms store and process the acquired acoustic signal data to prevent bio-diversity loss. This is not being done to identify how the use of cloud computing platforms negatively affects the environment.

Gadelha et al*.*^15^ address the challenge of biodiversity preservation in an ecosystem to quantify the biodiversity preservation concern. The considered variables are (1) Number of available species, (2) Number of individuals in each species and (3) Inter-species interaction context (trophic, competitive and symbiotic). In addition, the paper presents an informatics life cycle in addressing the biodiversity preservation challenge. The presented life cycle acquires data from sources such as videos, images, sounds, field trips data acquisition, remote sensing, publications, and biological sequences. The acquired data is analyzed using the approaches of machine learning; network-based modelling, string processing, computational modelling and data mining. The discussion also recognizes the relations between global changes such as dynamic patterns in land use evolution, increased atmospheric carbon dioxide and their influence on ecosystem processes. Ecosystem processes are described using species evenness, richness, interactions and composition.

A semantic approach is also presented by Gadelha et al*.*^15^ via an ecological metadata language. The ecological metadata language is developed to describe acquired ecological data i.e. species observation related datasets. The ecological metadata language considers information elements such as geographic coverage, temporal coverage, taxonomic coverage and sampling protocol. In addition, the ecological metadata language provides a base for developing metadata production and editing tools; and databases using repositories such as DataONE and web interfaces like Metacat. Newer formats such as the Darwin Core also present novel research perspectives on web-based technologies and their role in biodiversity preservation and monitoring.

The discussion in^15^ provides a review of different approaches such as network science and computational approaches that can be used for biodiversity preservation. The context of the presented research recognizes that progress is required from the perspective of acquiring data on different species and ensuring that the resulting datasets are shared globally with interested researchers. A techno-environmental analysis of a given technology alongside its evolution and environmental effects has not been considered.

From the discussion, it can be seen that the influence of cloud computing platforms on the environment is receiving research consideration. However, research in cloud computing largely follows two paths. The first is designing mechanisms to enhance cloud-based applications have improved quality of service^16–18^ and designing power-efficient data centre cooling systems^19,20^. A review of existing work in cloud computing platforms application with relations to environmental biodiversity is in Table 1. In addition, information on environmental concerns as it relates to variables and objectives are presented in Table 2.

**Table 1:** Review of existing work with relations to monitoring and preserving environmental bio-diversity.

Existing work	Contributions	Drawback
Kass et al*.*^2^	The discussion considers the challenges of data centre sustainable operation. This is done with relation to the effect on the environment. In addition, evolution in data centre systems is recognized as being important	The influence of paradigms such as free data centre cooling or reducing cooling costs has not been considered. In addition, work done in the Natick initiative has not been considered though future data centres receive research attention. Hence, non-terrestrial computing platforms are not considered
Brown^5^	The feasibility and benefits of using the ocean to host underwater data centres are recognized	The environmental effect of underwater data centres has not been explicitly considered. It is assumed that environmental requirements are satisfied before giving a permit
Bhattacherjee et al*.*^6^	Focus is on designing space based computing as a service to process data from a large number of low earth orbiting satellites deployed as a constellation. The computing as a service functionality is being offered as an additional service to the existing capability of network functionality. The added functionality is suited for applications generating their data in space	The environmental benefits and effects of the proposed computing as a service functionality has not been considered. The environmental benefits of using space-based computing edge nodes to share the computing load previously fully borne by terrestrial data centres have not received sufficient research consideration
Jones^9^	The discussion recognizes increasing data centre demand for electricity and water for cooling. A sustainable operation is considered from the perspective of reducing electricity and water consumption	The focus has been placed on using renewable energy sources to reduce the electrical energy demand to achieve sustainable data centre operation. This perspective of sustainable operations does not consider how data centre use influences habitat preservation
Facebook^10^	Approaches to realizing the sustainable operation of enterprise data centres are identified and discussed. The identified approaches are meeting the requirements of sustainability-related certifications; and the use of materials with low carbon impact	Additional discussion on the use of renewable energy sources and determining the renewable energy source that is suitable in different locations is required Furthermore, a list of potential server materials and their carbon impact has not been presented and will improve the presentation
Ganchev^14^	The presented research describes a network architecture enabling the acquisition and processing of acoustic signals from different habitats to ensure environmental bio-diversity	The discussion is set in the context of describing the role of data centres in computing platforms in preserving environmental bio-diversity. The context considers only terrestrial data centres. Non– terrestrial data centres have not been considered
Gadelha et al*.*^15^	The focus is surveying different methods that are used to evaluate and preserve bio-diversity. The role of computational methods is recognized	Focus here is on a survey and the objective of preserving environmental diversity and complying with cloud use policies has not been addressed
Jarke^21^	The role of cloud computing platforms in hosting industrial data is recognized in relation to international data space applications	The international data space application is being proposed in the context of using cloud platforms of data-driven industrial applications. However, the geo-environmental aspect of developing cloud applications has not received sufficient research consideration
Aydin et al*.*^22^	The focus of the discussion is on the realization of digital data sovereignty for the controlled storage and access of data	The discussion has not considered the underlying cloud platform architecture required to realize the proposed digital data sovereignty. In addition, the environmental impact in different locations due to the need to realize data sovereignty has not been considered
Celeste et al*.*^23^	The research addresses the challenge of data access in borderless cloud computing platforms. The distributed coverage of cloud computing platforms receives consideration. The difficulty of recognizing the most suitable regulation in accessing data is recognized	Significant challenges associated with ensuring environment-friendly operation of data centres in borderless cloud computing network architecture requires further research attention
Pham et al*.*^32^	The discussion presents a suite of digital and networking tools suitable for realizing the monitoring of urban biodiversity. The tools are database querying solutions, and network-related entities	The discussion has not explicitly considered how the deployment of cloud computing platforms influences environmental biodiversity
Chandler et al*.*^33^	The presented research describes the paradigm of citizen science. In the citizen paradigm, sensors are deployed with the aim of acquiring environment based variables.	The design of an integrated network architecture enabling the acquisition of environment based variables via wireless sensors deployed is required. However, this crucial subject requires additional research attention. In addition, the environment effect of data centre use also requires research consideration.

**Table 2:** Review of existing work with relations to data centre location and environmental variables.

Reference	Explicit data centre consideration	Data centre location	Considered variables and parameters
Kass et al.^2^	Yes	Terrestrial	Temperature, carbon footprint, water footprint, clean energy (renewable energy)
Roach^3^	Yes	Underwater	Temperature variation, humidity, renewable energy and sustainable strategies
Hume^4^	Yes	Underwater	Temperature, water use, water cooling, air cooling and cooling load
Brown^5^	Yes	Underwater	Renewable energy (tidal, wave energy), non use of temperature and other weather factors due to focus on the underwater environment
Bhattacherjee et al*.*^6^	Yes	Space	No focus on the use of earth climate. Space-based data centres do not utilize earth’s resources
Facebook^10^	Yes	Terrestrial	Circular thinking for e-waste reduction, open-source design to share efficiency design results, low environment impact server component materials, and renewable energy (wind, solar)
Google^11^	Yes	Terrestrial	Temperature monitoring, air cooling, water cooling, lighting control, carbon–neutral cloud services, renewable energy (carbon-free energy)
Ganchav et al*.*^14^	No (environmental biodiversity, terrestrial biodiversity and marine biodiversity receive consideration)		Bio-acoustics, eco-acoustics, terrestrial bio-diversity and marine bio-diversity monitoring
Gadelha et al.^15^	No	No	Environmental biodiversity description data are identified as species evenness, richness and composition Acquired environmental data are identified as videos, images, sound, land-use change and atmospheric carbon dioxide with the goal of aggregating environmental biodiversity variables Processing methods are identified as semantic web, network-based modelling, and deep learning

The discussion shows that there is an increasing concern on realizing data centre environment-friendly operation. The development and use of web-based tools^15^ are recognized to be suitable concerning realizing environment-friendly operation and habitat preservation. In addition, it is observed that computing metrics such as the WF and WUE require having access to data on water consumption which is not always available^1^. Hence, it is important to design a network architecture that reduces water usage and also devise a mechanism to proactively determine the use of water in a data centre.

**Figure 1:**
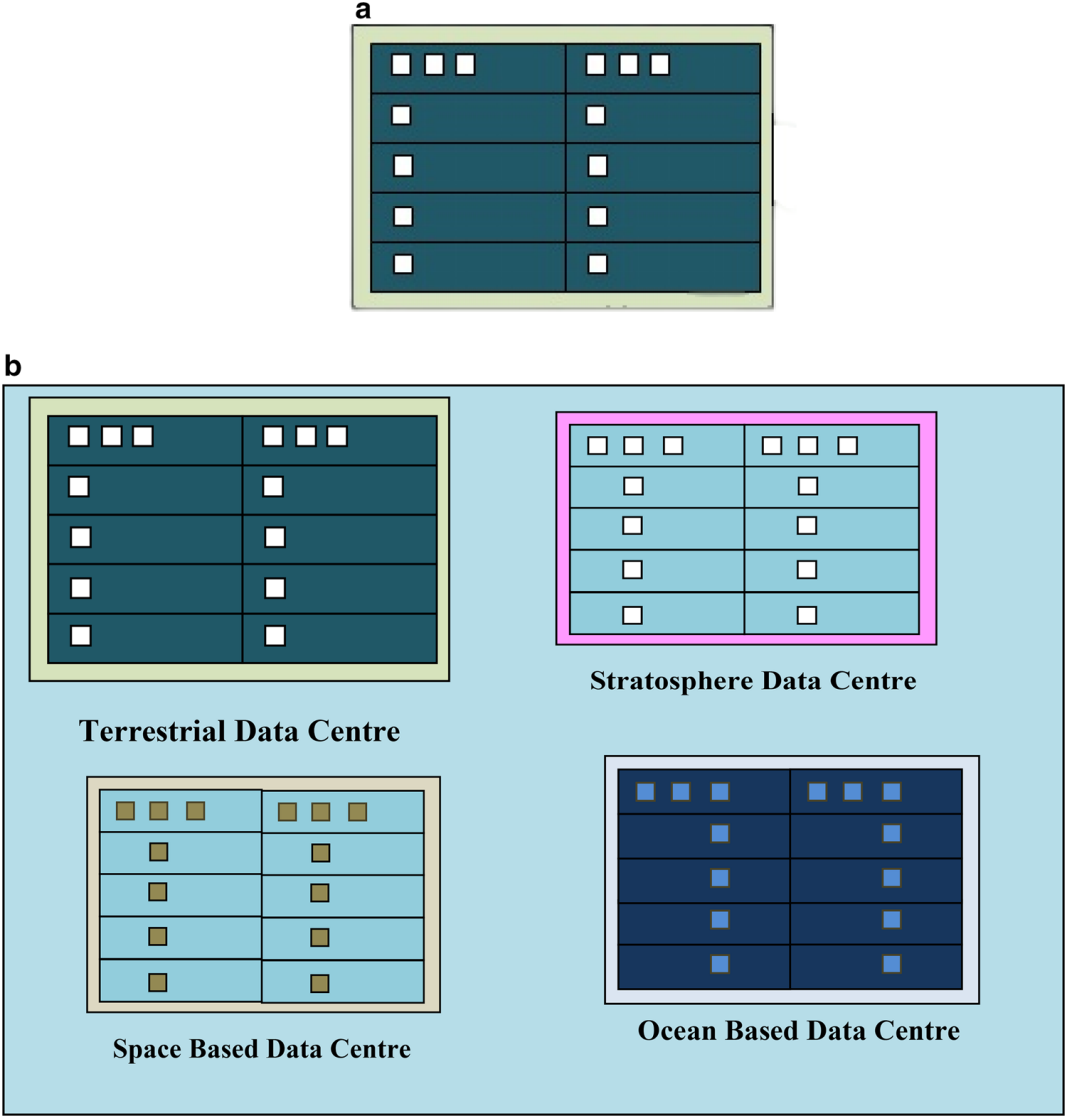
Resource usage depiction before and after considering hybrid cloud computing platforms. (**a**) Existing Approach with focus on usage of environmental resources (i.e. water for cooling) by terrestrial computing platforms only. (**b**) Hybrid cloud computing approach showing environmental resource usage (i.e. water) for cooling in a case comprising terrestrial and non-terrestrial data centres.

A description of the existing and proposed scenario is shown in Fig. 1a,b, respectively. Figure 1a shows the existing case with only terrestrial data centres. In this case, sustainability and environment-friendly operations are considered only for the terrestrial environment with a focus on urban areas (with sparse surroundings of biological habitats and maritime resources). Non-terrestrial data centres have not been considered. A sparse surrounding of maritime resources applies when terrestrial data centres are sited near resources like rivers, streams and lakes as seen in Google Hamina which is considered in^1^.

The case for the proposed mechanism is shown in Fig. 1b and considers terrestrial, stratosphere-based, space-based and underwater data centres. In Fig. 1b, stratosphere-based data centres are realized using high altitude platforms (HAPs). The motivation for the consideration of the stratosphere-based, space-based and underwater data centres stems from the need to reduce water usage as identified in^10^. This is because stratosphere-based and space-based data centres do not require water for cooling. In addition, their operation does not rely on grid-based electricity from the grid but electricity derived from the onboard solar energy system. The presence of stratosphere-based data centres necessitates the consideration of the avian habitat because the concerned data centres are located in the airspace. The use of space-based data centres is considered due to the non-use of water for cooling too. In addition, the stratosphere-based and space-based data centres do not rely on land thereby having a low land usage.

## Solution—realizing sustainability

The discussion here presents the proposed solution and has five aspects. The first aspect describes the proposed sustainable cloud computing platform model. The second aspect addresses the challenge of realizing global cloud computing platform environment-friendly operation. This is presented considering the short-term, medium-term and long-term realization. The third aspect presents a solution aiming to ensure that the cloud computing platform does not accelerate habitat extinction. This aspect considers the role of sensors and imaging technologies. The fourth aspect discusses the incorporation of support for data sovereignty and retention in the proposed architecture. The fifth aspect focuses on aspects relating to the proposed network architecture.

### Sustainability and the global cloud

Cloud platforms with varying capabilities can be realized via terrestrial and non-terrestrial computing entities that are placed in different locations. A capability classification of terrestrial and non-terrestrial computing platforms locations for a developing nation is presented in^24^. The classification in^24^ considers the capabilities and power system requirements of the different data centres in terrestrial and non-terrestrial locations. However, the realization of sustainable cloud computing systems aimed at preventing habitat extinction and compliance to data retention policies are not considered in^24^.

The proposed solution achieves environment-friendly operation of hybrid cloud computing systems while complying with data retention policies. The hybrid cloud computing systems comprise existing terrestrial and underwater data centres. These are used alongside space and stratosphere-based data centres.

**Figure 2:**
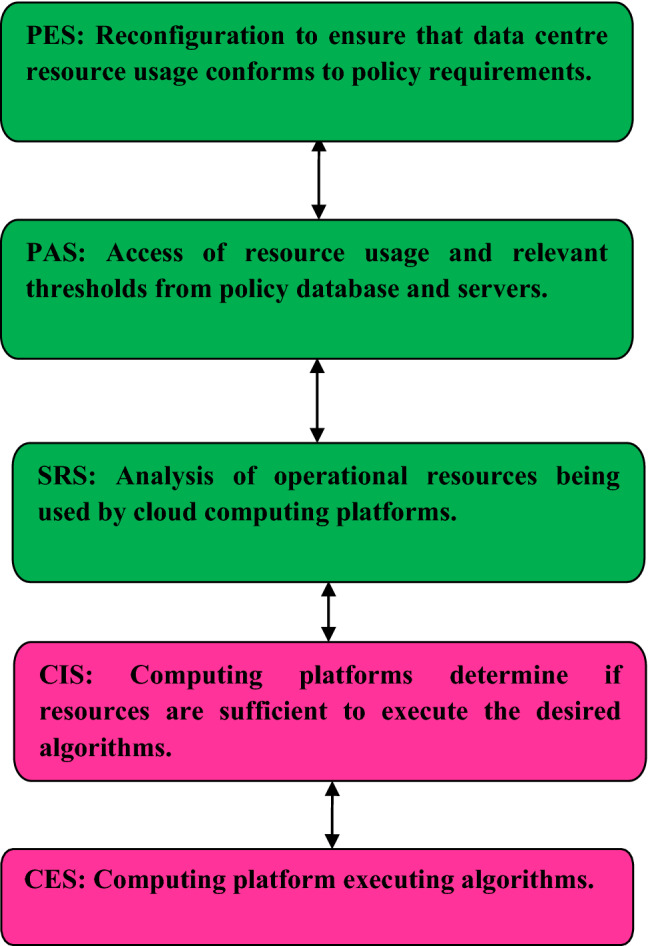
Relation between entities i.e. CES, CIS, SRS, PAS, and PES in the proposed solution.

The proposed multi-tier sustainable cloud computing model considers the realization of hybrid cloud systems comprising terrestrial and non-terrestrial cloud platforms. The proposed solution has five stages enabling environment-friendly operations. These are (1) Computing Execution Stage (CES), (2) Context Identification Stage (CIS), (3) Sustainability Requirement Stage (SRS), (4) Policy Access Stage (PAS), and (5) Policy Execution Stage (PES). The relation between the CES, CIS, SRS, PAS and PES is shown in Fig. 2. Figure 2 shows the tasks associated with the terrestrial data centre that is executed in the CES, CIS, SRS, PAS and PES. The scenario that has been presented is also applicable to non-terrestrial data centres. The terrestrial data centre executes data storage and computing algorithms in the CES. The terrestrial data centre receives workloads from computing platform subscribers. If the available computing resources are insufficient to process subscriber workloads, links are established with another data centre. This is determined in the CIS by the data centre cognitive algorithm (DCCA).

Figure 2 considers the case where external links are established with an underwater data centre having its own DCCA via the gateway (GW). The context is examined in the SRS to determine conformance to sustainable operational expectations. The DCCA monitors data centre resource usage in the SRS. This is done to ensure compliance with sustainability policy standards. The resource usage status about sustainability is accessed online by the DCCA from a policy database (PDb).

DCCAs of different data centres determine if reconfiguration is necessary after checking their compliance with the PDb content. The reconfiguration parameters required to ensure compliance are accessed from the reconfiguration database (RDb). Figure 2 shows the computing aspects that enable the realization of sustainable operations. This is done for a terrestrial and non-terrestrial data centre. However, it is important to consider multiple terrestrial and non-terrestrial data centres.

A contextual perspective to realizing sustainability is also considered. This is deemed necessary because of the inherent variable resource usage by data centres. Land usage effectiveness (LUE) is important in this regard. For a given land size, regions with a high population density need more land to meet accommodation demand in comparison to regions with a lower population density. The siting of data centres in high population density regions limits accommodation for the large human population. This does not arise in countries with a low population density.

Nevertheless, data centres should be sited in high population density regions to comply with data retention policies while realizing a low LUE and WF. In realizing a low LUE and WF in high population density regions, the use of aerial^7^, space-based^25–28^ and underwater data centres, are beneficial.

However, initiatives such as floating cities in^29–31^ may either compete with underwater data centres for access to the ocean or result in benefits. Nevertheless, the ocean’s vastness makes coastal cities in high population regions attractive for realizing non-cloud computing and cloud computing. Therefore, the use of non-terrestrial data centres (underwater and stratosphere) is suitable for high population regions. However, terrestrial data centres can be sited in regions with significant land sizes and low population density.

In addition, the proposed solution considers the realization of environmentally friendly operations while ensuring low LUE is realized in a phased approach. This phased approach enables the gradual incorporation of different data centres with varying levels of sustainable and environmentally friendly operations. The parameters of water footprint (WF) and water usage effectiveness (WUE) are utilized in the proposed approach. The WF and WUE are used to determine the transitions between the phases in the proposed mechanism. Each phase is accompanied with gradual addition of data centres whose operations have been deemed to achieve environmental sustainability and friendly operations.

Therefore, the consideration of phases in the short, medium and long terms incorporates the role of terrestrial and non-terrestrial data centres. The proposed global cloud computing platform comprises data centres from different cloud computing service providers. The server farms constitute a hybrid cloud computing platform comprising phases in the short, medium and long terms.

The execution of the short term phase involves requesting the consent of a selected number of cloud computing platforms (service providers) to participate in the hybrid cloud platform. The consenting providers donate servers to be used in realizing the hybrid cloud computing platform. In the medium term, more cloud computing service providers are considered. The progression to the medium-term phase occurs when sustainable operations i.e. reduced WF and WUE is realized in the short term phase. Further reduction in the WF and WUE in the medium term leads to the activation of the long term phase.

**Figure 3:**
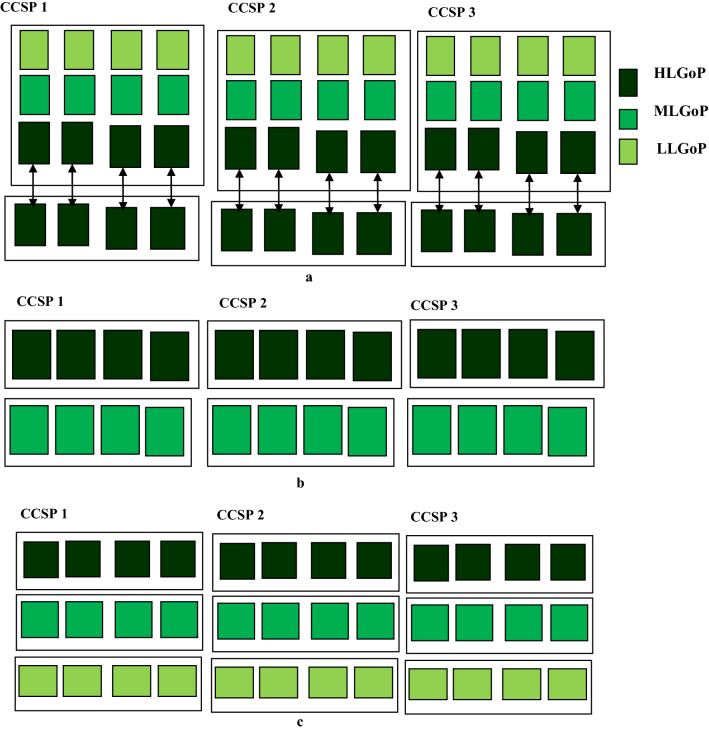
(**a**) Sustainable operation of hybrid cloud computing platform in the short term. (**b**) Sustainable operation of hybrid cloud computing platform in the medium term phase. (**c**) Sustainable operation achieved by the hybrid cloud computing platform in the long term phase.

The long term phase involves the full migration of selected cloud computing service providers to a hybrid computing platform. In this case, all server farms of the cloud service providers constitute the hybrid cloud computing platform. The procession to the long term phase indicates that WF and WUE have been enhanced in the short term and the medium term phases. The relation between the short term, medium-term and the long term phase are shown in Fig. 3a–c, respectively. The scenarios consider three cloud computing service providers (CCSPs), CCSP 1, CCSP 2, and CCSP 3. Each CCSP utilizes terrestrial, space-based, stratosphere-based, or underwater data centres.

From the perspective of sustainability, Fig. 3 has three data centres. These are high-level green operation (HLGoP), middle-level green operation (MLGoP) and low-level green operation (LLGoP). The data centres in these categories could be terrestrial data centres or non-terrestrial data centres (space-based, stratosphere-based and underwater data centres). The scenario in Fig. 3a–c show the realization of the hybrid cloud computing platform in the short term, medium-term and long term, respectively. It can be seen that server farms with improving environment-friendly operation are added from short term to long term through the medium-term phase.

### Computing platforms and sustaining habitats

Terrestrial data centres that are sited close to maritime resources use the nearby water resource for cooling. An increase in the maritime resource temperature occurs when warm water emerging from the data centre cooling process is released into the maritime resource. The resulting increase in the temperature of the maritime resource poses risks to aquatic biodiversity. Hence, reducing the WF reduces the risk being posed to aquatic bio-diversity. Underwater data centres when deployed in high numbers increase the surrounding ocean temperature which should not result in a degradation of aquatic bio-diversity. The monitoring of the temperature is executed by sub-surface temperature sensors. In the case of a significant increase in ocean temperature, the operation of one or more underwater data centres is unutilized or underutilised.

Terrestrial and underwater sensors enable monitoring an increase in temperature arising due to the release of warm water or warm air in underwater and terrestrial data centres. In these data centres, sensors are considered in the cases where the terrestrial data centres are close to life form hosting resources. The sub-surface temperature sensor sends an alert signal to the concerned data centre operator when the sub-surface temperature is noted to reach levels that are injurious to terrestrial and aquatic bio-diversity. In this case, a data centre that poses low risks to aquatic and terrestrial bio-diversity is selected for workload execution.

### Addressing data retention concerns

Data sovereignty is important for cloud computing platforms due to the need to address data access concerns between natural borders. The concept of data retention involves ensuring that access to data is managed and realized by obtaining consent from sovereign states. In this case, the ensuing data transfer proceeds when biodiversity is not threatened at the sites of the concerned data centres. In complying with data retention laws, it is important that corresponding data centres intending to share data with each other. This is established via the sustainable data centre pairing approach to ensure that cross-border data sharing is realized while ensuring environment-friendly data centre operation. The consideration in the underlying proposed sustainable data centre pairing approach applies to terrestrial, non-terrestrial and terrestrial to non-–terrestrial data centres connections.

Sustainable data centre pairing involves the analysis of biodiversity in the data centre locations. This is done for all data centre locations. The prior establishment of data retention compliance is conducted before executing biodiversity-related analysis (using wireless sensor network data). Data centre pairing also requires the establishment of a link between data centres communicating in a cross-border data exchange. This step involves getting the consent between CCSPs. Each consenting CCSP provides information on data source, applications, allowable countries, and biodiversity states.

The realization of data centre pairing requires the incorporation of capability enabling response to changes in data retention laws and policies. This is done via the execution of dynamic data retention analysis (DDRA) which is executed aboard the sustainability analysis centre (SAC). The DDRA determines how changing data retention policies affect the cloud platform link exchange stage. The DDRA ensures compliance with changing data sovereignty policies. The SAC receives information on policies via the internet. The transmission session is initiated from a government-owned facility in a sovereign state. A change in the SAC’s content triggers a re-examination of cloud platform link establishment.

The concerns of biological conservation are accommodated via the conservation and observation window. The conservation window enables the biology conservation community to access bio-diversity information via the SAC. The access enables the biological conservation interests to access information on the influence of computing platforms on the environment. The conservation window also enables the biology conservation organisation to change the biodiversity compliancy requirements.

**Figure 4:**
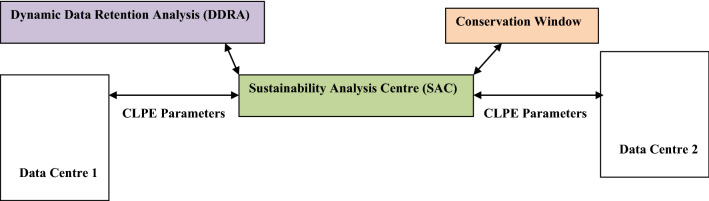
Relations between two data centres, SAC, changing retention policies and conservation interests (conservation window).

The relation between the execution of the cloud platform link establishment, DDRA and the conservation window is shown in Fig. 4. Figure 4 shows the parameter exchange in the cloud platform link exchange (CPLE) stage. The presented scenario involves three entities. These are Data Centre 1, Data Centre 2 and the SAC. The SAC is placed in a buffer zone i.e. a location with low cybercrime incidences (due to robust cyber-security solutions deployment). The buffer zone receives data from the participating sovereign states. The scenario shows a case involving the data centres in two sovereign states. However, a concurrent relation between multiple states and SACs is also feasible. In Fig. 4, the DDRA enables several sovereign states to update their data retention policies in the SAC. The conservation window enables biological interests to upload changing preferences on biological conservation to the SAC. It also enables the biological conservation research interests to obtain habitat friendly related information via the wireless sensor network linked to the SAC via data centres.

### Enabling government policies required for sustainable system realization

The proposed solution supports data retention and habitat conservation interests. The required networking infrastructure is realized via internet exchange points. This is feasible as different countries already have an internet exchange point^34–36^ that are used for enforcing data retention policies. The sharing of data occurs under new computing constructs as data sharing can only occur between computing platforms in different states if the computing platforms store data and suitably execute algorithms. The verification of this new construct is executed by the SAC in the buffer zone. This is done while considering evolving governmental policies. The role of policies from global bodies such as the United Nations Environment world conservation monitoring centre such as^37–39^ is also considered.

### System realization and network integration

**Figure 5:**
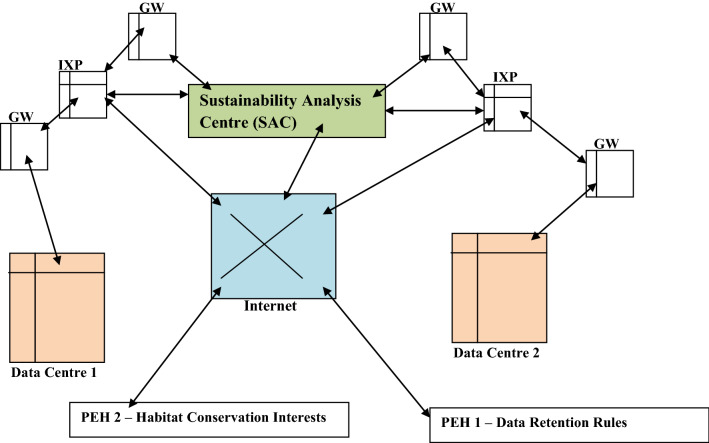
Novel Network Architecture showing relations between entities enabling sustainable operation.

The proposed approach is implemented as a networked solution. The proposed network architecture comprises internet exchange points (IXPs), data centres, computing entities and gateways (GWs). In the network architecture, data centres store and process data. The relation between the GW, IXP, policy evolution handlers (PEHs), data centres 1 and 2 is presented in Fig. 5. PEH 1 and PEH 2 receive information on policy evolution from the government and biological conservation, respectively.

**Figure 6:**
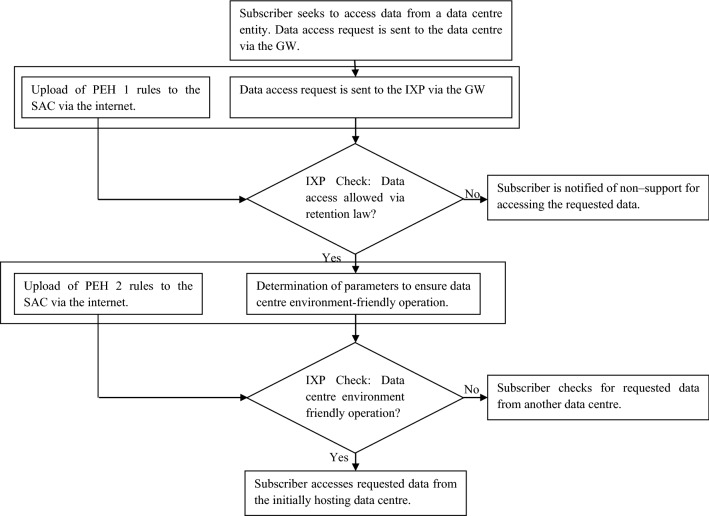
Flowchart showing the relation between entities enabling the proposed solution.

In addition, a flowchart showing the steps being executed in the network architecture shown in Fig. 5 has been presented in Fig. 6. The flowchart in Fig. 6 considers the case of a subscriber seeking data access. There are two data centres i.e. terrestrial data centre TDC 1 and underwater data centre UDC 1. In this case, the data being requested for access is hosted aboard TDC 1. The policies regarding data retention and habitat conservation are uploaded to the SAC via the internet and accessible at a regional IXP and GW. TDC 1 (that satisfies habitat conservation goals) enables the subscriber to access the requested data given supporting data retention policies. If the use of TDC 1 does not meet habitat conservation goals, the data access request is not supported though data retention policies support the concerned access. In this case, the use of another data centre i.e. underwater data centre UDC 1 with support for habitat conservation and data retention enabled access is preferred. In the absence of the proposed approach, the requested data is accessed (with supporting data retention laws) without consideration of the need to ensure environment-friendly operation (if habitat conservation goals are not supported). However, if there is strict enforcement of habitat conservation goals, the requested data is not accessed by the requesting subscriber. In this case, there is no alternative data centre that can provide access to the requested data. The differences between realizing environment sustainability for the existing and proposed approach is shown in Table 3.

**Table 3:** Comparison between existing and proposed approaches from an environmental sustainability perspective.

S/N	Metric	Existing approach	Proposed approach
1	Terrestrial Habitat Extinction	Terrestrial data centres in urban areas do not pose threats to biodiversity as they are sited far from forests and game reserves	The terrestrial data centre component in hybrid data centres doesn’t pose threats to biodiversity when distant from forests and game reserves
2	Marine Habitat Influence	Water-cooled terrestrial data centres sited close to maritime resources can have potentially high levels of thermal anthropogenic effect. This is only applicable to terrestrial data centres that are sited close to maritime resources and use the associated water resources for low-cost cooling. An example is Google Hamina^1^	Underwater data centres are integral in the hybrid cloud data centre. This causes higher levels of thermal pollution in the ocean’s vicinity of underwater data centres. The challenge arises when a high number of underwater data centres are deployed. This is applicable when marine heat waves occur. The challenge is yet to receive sufficient consideration
3	Avian Habitat Influence	Terrestrial data centre operation does not pose threats to avian life	Aerial data centres in the hybrid cloud data centre pose threats to avian life
4	Existing Computing Entities	Terrestrial Data Centres	Aerial, Terrestrial, Underwater and Space-Based Data Centres
5	Technology Deployment	Terrestrial Data Centre technology has been widely used	Non-terrestrial data centre technologies are yet to be widely deployed and are in the testing phase
6	Data Retention and Allowable Sharing Locations	The implementation of data retention laws for terrestrial data centres requires defined land borders for considered locations	Implementation of data retention laws concerns land borders, air-space and ocean regions for terrestrial, aerial and underwater data centres, respectively
7	Computing Component Cooling	Free air-cooling, Air-cooling, cold water cooling and warm water cooling,	Free air-cooling, Air-cooling, cold water and warm water cooling. Stratosphere cooling for stratosphere data centres. Ocean water for underwater data centres
8	Network Architecture	Existing network architecture focuses on realizing sustainable operations via renewable energy such as wind and solar technologies	The proposed network embraces the use of renewable energy sources alongside evolving preferences of biological conservation research
9	Cross-Border Data Exchange Process	Cross-border data exchange between data centres in multiple locations is subject to the data retention policies of concerned countries	Data centres engaged in cross-border data exchange comply with sovereign data retention laws and preferences of biological conservation
10	Land Usage and Reliance on Land	Terrestrial Data Centres rely on having access to land and reduce the land accessible for other applications	A reduction in land usage is achieved by placing data centres in the stratosphere and the ocean alongside terrestrial data centres
11	Use of earth’s water resources	Water-cooled data centres have a significantly high level of reliance on the earth’s water resources	The earth’s water resource is used by underwater data centres without affecting water access for agriculture. Space-based and stratosphere-based data centres don’t rely on earth’s water resources for cooling

In Table 3, the existing approach describes research in^1,5,9,10^. The research in^5^ recognizes the suitability of using water for cooling submerged data centres and doesn’t consider a rise in water level with the use of more underwater computing platforms. The discussion in^9^ recognizes that satisfying the energy and water usage demand of terrestrial data centres poses a challenge. The approach in^10^ considers Facebook’s work in realizing sustainable data centre operations. The focus in^10^ is wholly on terrestrial data centres. The work in^1,5,9,10^ focuses on a context involving terrestrial and underwater data centres only. The proposed mechanism considers a context comprising terrestrial, stratosphere-based and underwater data centres.

## Performance formulation

The WP is formulated in this section. The WP differs from the existing metrics of the water footprint (WF) and the water usage effectiveness (WUE). The WF and WUE are evaluated to determine the water consumption of existing data centres. These metrics i.e. WF^1,7^ and WUE^9,10^ are evaluated for terrestrial data centres. They are not examined for space-based and stratosphere-based data centres that do not directly use water for server cooling. Being based in the underwater environment, underwater data centres utilize water for cooling. However, the WF and WUE are insufficient to consider the effect of underwater data centre cooling on the use of water. This is because underwater data centres do not experience water constraints like terrestrial data centres. Nevertheless, deploying underwater data centres in large numbers make significant use of water in the ocean environment.

The WF and WUE are not suitable for considering relations between underwater data centre cooling and water usage. This applies to space-based data centres and stratosphere-based data centres. In the case of water-cooled space-based data centres and stratosphere-based data centres, the amount of water that may be utilized in a potential cooling procedure has not been potentially evaluated to negatively influence on earth’s rainfall. However, the water consumption potential of these data centres is important for future consideration when deployment may increase. A metric that considers water usage from this perspective is required. The WP metric is proposed in this regard. This is because the WF and WUE are not defined for the concerns arising in the case of underwater, space-based and stratosphere-based data centres. The WF and WUE are defined to estimate the water usage for terrestrial data centres. The parameters that are used in this section alongside their rationale are shown in Table 4.

**Table 4:** List of Notations and Parameters.

S/N	Parameter	Description and rationale
1	${\alpha }_{T}$	Set of terrestrial data centres (water-cooled). The set of terrestrial data centres have been considered with a view to formulating the water potential (WP) and the role of water in data centre operations with regards to cooling
2	${\alpha }_{S}$	Set of stratosphere based data centres. The set of stratosphere based data centres has been considered as a non-terrestrial data centre that does not require water for cooling
3	${\alpha }_{s}$	Set of space-based data centres. The set of space-based data centres has been considered as a non-terrestrial data centre that does not require water for cooling
4	${\alpha }_{O}$	Set of underwater data centres. The set of underwater data centres has received consideration as a non-terrestrial i.e. data centres that do not require terrestrial water resources for cooling
5	${\alpha }_{T}^{a},{\alpha }_{T}^{a} \epsilon {\alpha }_{T}$	The ${a}\mathrm{th}$ terrestrial data centre. The role of each terrestrial data centre has been considered to demonstrate that each terrestrial data centre utilizes water for cooling
6	${\alpha }_{S}^{b}, {\alpha }_{S}^{b} \epsilon {\alpha }_{S}$	The ${b}\mathrm{th}$ stratosphere based data centre. The role of each stratosphere data centre has been considered to demonstrate that the use of each stratosphere based data centre (non-terrestrial data centre) results in a reduced water footprint i.e. the demand of water for water centre cooling
7	${\alpha }_{s}^{c}, {\alpha }_{s}^{c} \epsilon {\alpha }_{s}$	The ${c}\mathrm{th}$ space-based data centre. The role of each space data centre has been considered to demonstrate that the use of each space-based data centre (non-terrestrial data centre) results in a reduced water footprint i.e. the demand of water for water centre cooling
8	${\alpha }_{O}^{d},{\alpha }_{O}^{d} \epsilon {\alpha }_{O}$	The ${d}\mathrm{th}$ underwater data centre. The role of each underwater data centre has been considered to demonstrate that the use of each underwater data centre (non – terrestrial data centre) results in a reduced water footprint i.e. demand of water for data centre cooling. This is because of the reduced demand for terrestrial water resources
9	$N\left({\alpha }_{T}^{a}\right)$	The number of stages in the cooling process for the water-cooled ${a}\mathrm{th}$ terrestrial data centre. This variable has been introduced to demonstrate the different number of stages associated with cooling in terrestrial data centre (due to different technologies i.e. heat exchangers)
10	$N\left({\alpha }_{S}^{b}\right)$	The number of stages associated with the realization of cooling in a stratosphere based data centre i.e. the ${b}\mathrm{th}$ stratosphere based data centre. This variable is used to demonstrate that the stratosphere based centre can have a varying number of stages in the heat exchangers in cooling the computing payload
11	$N\left({\alpha }_{s}^{c}\right)$	The number of stages associated with the realization of cooling in a space data centres i.e. the ${c}\mathrm{th}$ space data centre. This variable is used to demonstrate that space data centres can have a varying number of stages i.e. the number of heat exchangers in cooling the computing payload
12	$N\left({\alpha }_{O}^{d}\right)$	The number of stages associated with the realization of cooling in an underwater data centre i.e. the ${d}\mathrm{th}$ underwater data centre. This variable is used to demonstrate that each underwater data centre can have a varying number of heat exchanger stages used in cooling the computing payload
13	${t}_{y}$	The ${y}\mathrm{th}$ epoch is considered in the set of time instants. The rationale for using this variable is to describe our consideration for a time-varying behaviour of the considered computing platforms
14	$I\left({\alpha }_{T}^{a},{t}_{y}\right)$	The cooling status of each data centre with a focus on the terrestrial data centre. The variable is used with to demonstrate that terrestrial data centres can either be water-cooled or air-cooled at the considered ${y}\mathrm{th}$ epoch

The WP is used to quantify the perceived need of a data centre or a computing platform to require water for cooling. This metric is defined in the planning phase and is different from the WF and WUE. The WF and WUE are only evaluated after the concerned computing platform has used water for its cooling. The WP is estimated during the data centre or computing platform design phase.

Let ${\alpha }_{T} , {\alpha }_{S}$ , ${\alpha }_{s}$ and ${\alpha }_{O}$ be the set of terrestrial, space-based, stratosphere-based and ocean-based data centres, respectively. Terrestrial data centres can be either water-cooled or air-cooled.

1 $${\alpha }_{T}=\left\{{\alpha }_{T}^{1}, {\alpha }_{T}^{2},\dots , {\alpha }_{T}^{A}\right\}$$

2 $${\alpha }_{S}=\left\{{\alpha }_{S}^{1}, {\alpha }_{S}^{2},\dots , {\alpha }_{S}^{B}\right\}$$

3 $${\alpha }_{s}=\left\{{\alpha }_{s}^{1}, {\alpha }_{s}^{2},\dots , {\alpha }_{s}^{C}\right\}$$

4 $${\alpha }_{O}=\left\{{\alpha }_{O}^{1}, {\alpha }_{O}^{2},\dots , {\alpha }_{O}^{D}\right\}$$

where: $A, B, C, D$ are the indexes of the last terrestrial, space-based, stratosphere-based and ocean-based data centres, respectively. This is the total number of data centres in each considered location.

Let $I\left({\alpha }_{T}^{a},{t}_{y}\right) \epsilon \{\mathrm{0,1}\}, {\alpha }_{T}^{a} \epsilon {\alpha }_{T} ,{t}_{y}\epsilon t, t=\{{t}_{1},\dots , {t}_{Y}\}$ denote the cooling status of the ${a}\mathrm{th}$ terrestrial data centre ${\alpha }_{T}^{a}$ at the ${y}\mathrm{th}$ epoch ${t}_{y}$. The data centre ${\alpha }_{T}^{a}$ is water-cooled and air-cooled at the epoch ${t}_{y}$ when $I\left({\alpha }_{T}^{a},{t}_{y}\right)=1$ and $I\left({\alpha }_{T}^{a},{t}_{y}\right)=0$, respectively. The number of stages in the entity $q , q \epsilon \left\{{\alpha }_{T}^{a},{\alpha }_{S}^{b} ,{\alpha }_{s}^{c},{\alpha }_{O}^{d}\right\} , {\alpha }_{S}^{b} \epsilon {\alpha }_{S} , {\alpha }_{s}^{c} \epsilon {\alpha }_{s} , {\alpha }_{O}^{d} \epsilon {\alpha }_{O}$ is denoted $N(q)$.

The WP is formulated for the case of the existing mechanism and the proposed mechanism. The WP is formulated for the proposed mechanism considering different phases in which data centres in different locations are considered. This enables a formulation of the WP for the proposed mechanism. The formulation in this manner enables an investigation of the WP given cases where multiple data centres are considered in the realization of the proposed solution.

The WP is formulated for scenarios comprising a total of $A$ data centres that are operational for a duration spanning the epochs between the time instants ${t}_{1}$ and ${t}_{Y}$. The WP in the existing scenario $\dot{\Gamma }_{1}$ is given as:

5 $${\dot{\Gamma }}_{1}= \sum_{a=1}^{A}\sum_{y=1}^{Y}I\left({\alpha }_{T}^{a},{t}_{y}\right)N\left({\alpha }_{T}^{a}\right)$$

The relation in (5) i.e. the WP formulated and presented in ${\dot{\Gamma }}_{1}$ concerns the case of existing solutions. In this case, hybrid computing platforms have not been considered. Hence, the role of non-terrestrial computing platforms (located in space, stratosphere or underwater) have not been considered. Terrestrial data centres can be either water-cooled or air-cooled.

The WP in the case comprising terrestrial and space-based data centres (proposed case-hybrid computing platforms) is denoted ${\dot{\Gamma }}_{2}$ and given as:

6 $${\dot{\Gamma }}_{2}=\sum_{y=1}^{Y}\sum_{a=1}^{A-p}\sum_{b=1}^{p}\left(I\left({\alpha }_{T}^{a},{t}_{y}\right)N\left({\alpha }_{T}^{a}\right)+ N\left({\alpha }_{S}^{b}\right)\right), {\alpha }_{S}^{p}\epsilon {\alpha }_{S} , p\le B$$

The case in Eq. (6) considers that there are a total of $p$ space-based data centres. In this case, given that there are $A$ total data centres in the consideration, there are $A-p$ terrestrial data centres.

The WP in a case comprising terrestrial and stratosphere-based data centres is denoted ${\dot{\Gamma }}_{3}$ and given as:

7 $${\dot{\Gamma }}_{3}=\sum_{y=1}^{Y}\sum_{a=1}^{A-p}\sum_{c=1}^{p}\left(I\left({\alpha }_{T}^{a},{t}_{y}\right)N\left({\alpha }_{T}^{a}\right)+ N\left({\alpha }_{s}^{c}\right)\right),{\alpha }_{s}^{p}\epsilon {\alpha }_{S} , p\le C$$

The case in Eq. (7) considers that there are a total of $p$ stratosphere-based data centres. In this case, given that there are $A$ total data centres in the consideration, there are $A-p$ terrestrial data centres.

In the case comprising terrestrial and underwater-based data centres, the WP, ${\dot{\Gamma }}_{4}$ is:

8 $${\dot{\Gamma }}_{4}= \sum_{y=1}^{Y}\sum_{a=1}^{A-p}\sum_{d=1}^{p}\left(I\left({\alpha }_{T}^{a},{t}_{y}\right)N\left({\alpha }_{T}^{a}\right)+ N\left({\alpha }_{O}^{d}\right)\right),{\alpha }_{O}^{p}\epsilon {\alpha }_{S} , p\le D$$

The WP in Eq. (8) is formulated for a scenario comprising terrestrial and underwater-based data centres. In this case, there are a total of $p$ ocean-based data centres. In this case, given that there are $A$ total data centres in the consideration, there are $A-p$ terrestrial data centres.

The proposed scenario can also involve a case excluding terrestrial data centres. In this case, the WP ${\dot{\Gamma }}_{5}$ is:

9 $${\dot{\Gamma }}_{5}=\sum_{b=1}^{A-2p}\sum_{c=1}^{p}\sum_{d=1}^{p}\left(N\left({\alpha }_{S}^{b}\right)+N\left({\alpha }_{s}^{c}\right)+N\left({\alpha }_{O}^{d}\right)\right), {\alpha }_{S}^{A-2p}\epsilon {\alpha }_{S} , A>3p , p\ge 0, {\alpha }_{s}^{p}\epsilon {\alpha }_{s} ,{\alpha }_{O}^{d}\epsilon {\alpha }_{O}$$

In Eq. (9), the considered scenario comprises ocean-based, space-based and stratosphere-based data centres. The formulation comprises $p$ underwater data centres, $p$ stratosphere-based data centres and $A-2p$ space-based data centres.

## Performance simulation and evaluation

The results for the WP are presented in this section. This is done using the parameters in Table 5. Table 5 shows the stages requiring water for data centres in the concerned locations. The total number of data centres in each location i.e. terrestrial, stratosphere-based, space-based and underwater data centres used in the simulation is presented in Table 6.

**Table 5:** Number of stages requiring the use of water in the concerned data centre locations.

Parameter	Description	Value
The number of stages requiring water in terrestrial data centres	The stage involves (1) Chillers, (2) Pumps, (3) Circulators, (4) Internal Heat Exchangers and (5) External Heat Exchangers	5
The number of stages requiring water in cooling stratosphere based data centres	Stratosphere based data centre relies on the effect of stratospheric cooling and makes use of water via the Pumps, and External Heat Exchangers	2
The number of stages requiring water in cooling space data centres	Space data centres don’t rely on earth’s water resources	0
The number of stages requiring water in cooling underwater based data centres	The stage involves (1) Pumps, (2) Circulators, (3) Internal Heat Exchangers and (4) External Heat Exchangers	4

**Table 6:** Number of data centres in considered locations.

**S/N**	Parameter	Value
**Total number of terrestrial data centres–**${\varvec{A}}$		
1	Maximum Number of water-cooled terrestrial data centres	551
2	Minimum Number of water-cooled terrestrial data centres	21
3	Mean Number of water cooled terrestrial data centres	280
**Total number of stratosphere data based data centres. This is the variable** ${\varvec{p}}$		
4	Maximum number of stratosphere-based data centres	504
5	Minimum number of stratosphere-based data centres	3.1
6	Mean number of stratosphere-based data centres	255
**Total number of data centres in space (**${\varvec{p}}$**)**		
7	Maximum Number of Data Centres in Space	469
8	Minimum Number of Data Centres in Space	18
9	Mean Number of Data Centres in Space	237
**Total number of underwater-based data centres (**${\varvec{p}}$**)**		
10	Maximum Number of underwater-based Data Centres	46
11	Minimum Number of underwater-based Data Centres	1.6
12	Mean Number of underwater-based Data Centres	25

The performance evaluation is done for two contexts. In the first context, the WP is evaluated for the hybrid cloud computing platforms considering the different combination of terrestrial data centres and non-terrestrial data centres (i.e. space-based, stratosphere-based or underwater data centres). The second context evaluates the WP for a case comprising only terrestrial data centres. However, the terrestrial data centres are either water-cooled or air-cooled. In this case, different proportions of air-cooled terrestrial data centres and water-cooled terrestrial data centres are considered.

**Figure 7:**
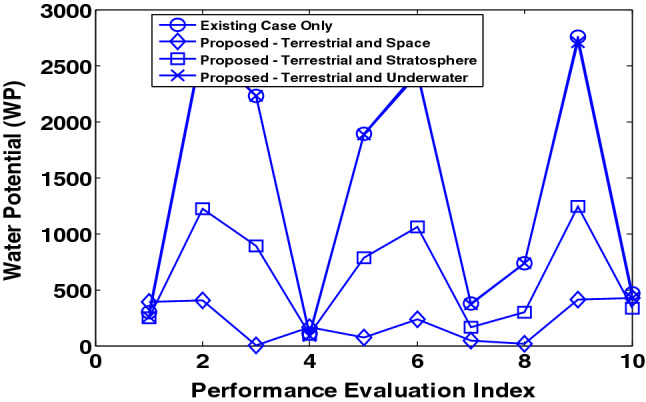
WP for the existing scheme and proposed scheme (in different contexts).

**Figure 8:**
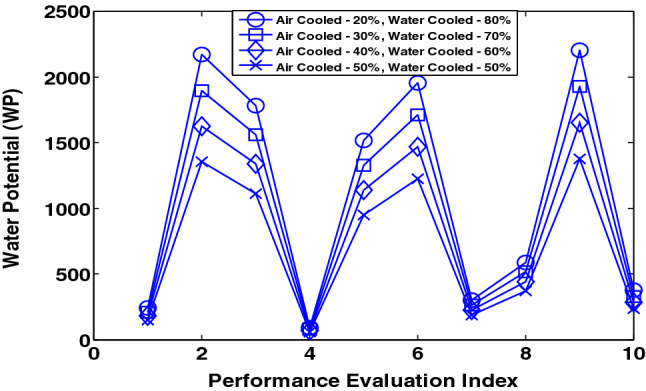
WP for the existing terrestrial data centres with different proportion of air-cooled and water-cooled systems.

The investigation to evaluate the WP is done using the parameters in Tables 3 and 4. The results obtained for the WP for terrestrial water-cooled data centres and data centres in the stratosphere, outer-space and underwater environment is shown in Fig. 7. The evaluation also investigates the WP for cases comprising air cooled and water cooled terrestrial data centres. The results, in this case, are in Fig. 8.

Figure 7 shows that the WP is low for data centres in the stratosphere, space and underwater environment in comparison to terrestrial data centres (water-cooled). This is because of the use of water in five stages in water-cooled terrestrial data centres. Analysis shows that using stratosphere-based, space-based and underwater-based data centres alongside terrestrial data centres in comparison to existing mechanism (only terrestrial data centres) reduces the WP by 45.4%, 57% and 4.9% on average, respectively.

In addition, the performance evaluation considers more cases with a varying number of data centres in terrestrial, stratosphere, outer-space and underwater locations. The evaluation parameter for 10 additional epochs is shown in Tables 7 and 8. The performance benefit i.e. reduction in water potential (WP) due to the variation in the number of data centres is evaluated for 10 additional simulation epochs is shown in Table 9.

**Table 7:** System Model Parameters for the first seven epochs.

S/N	Parameter	Epoch 1	Epoch 2	Epoch 3	Epoch 4	Epoch 5	Epoch 6	Epoch 7
1	Maximum Number of Terrestrial Data Centres	181	171	193	181	143	122	66.5
2	Minimum Number of Terrestrial Data Centres	20	9.5	40.4	19.5	9.75	3.45	13.3
3	Mean Number of Terrestrial Data Centres	90	104	120.2	103.3	76.6	77.4	45.5
4	Maximum number of Stratosphere Data Centres	152	147	188	175	140	116.4	63.2
5	Minimum number of Stratosphere Data Centres	0.8	2.6	38.5	7.7	7.6	0.996	8.99
6	Mean number of Stratosphere Data Centres	64.9	80.5	113.8	96.8	70.7	74.8	41.4
7	Maximum Number of Data Centres in Space	149	128	188.3	176	140.5	116.8	63.4
8	Minimum Number of Data Centres in Space	29.4	22.8	38.6	8.2	7.7	1.2	9.3
9	Mean Number of Data Centres in Space	46	61.9	114.1	97	71	74.94	41.7
10	Maximum Number of Underwater Data Centres	49	41.4	36	49.3	47.8	46.4	49.8
11	Minimum Number of Underwater Data Centres	2.7	6.9	7.8	0.23	7.7	2.6	1.8
12	Mean Number of Underwater Data Centres	26	24.4	26.5	27.5	24.4	19	29.5

**Table 8:** System model parameters for the last three epochs.

S/N	Parameter	Epoch 8	Epoch 9	Epoch 10
1	Maximum Number of Terrestrial Data Centres	115	35.3	56.0
2	Minimum Number of Terrestrial Data Centres	14.4	0.53	4.93
3	Mean Number of Terrestrial Data Centres	79.4	24	31.6
4	Maximum number of Stratosphere Data Centres	111.2	35.3	51.2
5	Minimum number of Stratosphere Data Centres	11.4	0.53	1.66
6	Mean number of Stratosphere Data Centres	75.3	24	28.6
7	Maximum Number of Data Centres in Space	111.5	35.6	51.2
8	Minimum Number of Data Centres in Space	11.6	1	1.9
9	Mean Number of Data Centres in Space	75.6	24.3	28.8
10	Maximum Number of Underwater Data Centres	47.8	47.9	44.4
11	Minimum Number of Underwater Data Centres	17.7	2.6	3.4
12	Mean Number of Underwater Data Centres	29.5	28.2	21.4

**Table 9:** Performance benefit (reduction in WP) in all epochs.

Parameter	Epoch 1	Epoch 2	Epoch 3	Epoch 4	Epoch 5	Epoch 6	Epoch 7	Epoch 8	Epoch 9	Epoch 10
Mean Reduction in WP (Space Data Centres) %	23	42	95	87	89	90	89	93	81	85
Mean Reduction in WP (Stratosphere Data Centres) %	34	40	57	52	53	54	53	56	48	51
Mean Reduction in WP (Underwater Data Centres) %	8.8	7	5	11	9	15	16	11	29	23

From the presented results, it can be seen that the use of stratosphere-based, space-based and underwater-based data centres alongside terrestrial data centres in comparison to the existing mechanism (only terrestrial data centres) reduces the WP by (34–56) %, (23–93) %, and (4.9–23) % on average, respectively.

The results in Fig. 8 show the WP obtained in the case where varying proportions of data centres are either water-cooled or air-cooled. The proportion of data centres that are air-cooled and water-cooled is presented as $\{x, y\}$. The WP in the existing case i.e. with only water-cooled terrestrial data centres in comparison to the cases $\left\{20\mathrm{\% }, 80\mathrm{\%}\right\}, \{30\mathrm{\% }, 70\mathrm{\%}\}$, $\{40\mathrm{\% }, 60\mathrm{\%}\}$ and $\{50\mathrm{\% }, 50\mathrm{\%}\}$ is reduced by an average of 20%, 30%, 40% and 50%, respectively. Therefore, reducing the number of water-cooled terrestrial data centres and the use of more air-cooled non-terrestrial data centres reduces the water footprint.

## Conclusion

The presented research proposes and presents novel network architecture aimed at ensuring the sustainable operation of hybrid computing platforms. Hybrid computing platforms comprise terrestrial data centres and non-terrestrial data centres i.e. underwater (submarine), stratosphere-based and space-based data centres. The paper also recognizes that the data centre usage poses anthropogenic effects (thermal pollution). The use of water for data cooling and utilization of certain locations hosting maritime resources also receives consideration. Thermal pollution arises from the release of heat into the environment due to the occurrence of data centre cooling. The resulting pollution causes biodiversity degradation. The discussion addresses the prevention of habitat biodiversity degradation via computing platform utilization. Furthermore, the reliance on water poses a significant concern when a significant number of data centres are deployed to meet the increasing needs for data storage and processing. The proposed architecture aims to consider the interests of biological conservation and water security. It describes how networking entities interact to realize the presented cloud-habitat sustainable cyber-physical computing system. In addition, the paper evaluates the performance of the proposed network architecture using the newly considered water potential metric. The metric of water potential is proposed and used in a novel manner that considers the future water demand and usage for data centres in the considered hybrid computing platform environment. The water metric should be reduced because its low value shows that the concerned computing platform requires less water for its cooling processes. The evaluation shows that the water potential metric is enhanced by the use of the proposed network architecture.
